# A map of white matter tracts in a lesser ape, the lar gibbon

**DOI:** 10.1007/s00429-023-02709-9

**Published:** 2023-10-31

**Authors:** Katherine L. Bryant, Paul R. Manger, Mads F. Bertelsen, Alexandre A. Khrapitchev, Jérôme Sallet, R. Austin Benn, Rogier B. Mars

**Affiliations:** 1 Wellcome Centre for Integrative Neuroimaging, Centre for Functional MRI of the Brain (FMRIB), Nuffield Department of Clinical Neurosciences, John Radcliffe Hospital, University of Oxford, Oxford, UK; 2 Laboratoire de Psychologie Cognitive, Aix-Marseille Université, Marseille, France; 3 School of Anatomical Sciences, Faculty of Health Sciences, University of Witwatersrand, Johannesburg, South Africa; 4 Centre for Zoo and Wild Animal Health, Copenhagen Zoo, Frederiksberg, Denmark; 5 Department of Oncology, University of Oxford, Oxford, UK; 6 Stem Cell and Brain Research Institute, Université Lyon 1, Inserm, Bron, France; 7 Integrative Neuroscience and Cognition Center, Université de Paris, CNRS, Paris, France; 8 Donders Institute for Brain, Cognition and Behaviour, Radboud University, Nijmegen, The Netherlands

**Keywords:** Fasciculus, Tractography, DWI, Hominoid, Evolution

## Abstract

The recent development of methods for constructing directly comparable white matter atlases in primate brains from diffusion MRI allows us to probe specializations unique to humans, great apes, and other primate taxa. Here, we constructed the first white matter atlas of a lesser ape using an *ex vivo* diffusion-weighted scan of a brain from a young adult (5.5 years) male lar gibbon. We find that white matter architecture of the gibbon temporal lobe suggests specializations that are reminiscent of those previously reported for great apes, specifically, the expansion of the arcuate fasciculus and the inferior longitudinal fasciculus in the temporal lobe. Our findings suggest these white matter expansions into the temporal lobe were present in the last common ancestor to hominoids approximately 16 million years ago and were further modified in the great ape and human lineages. White matter atlases provide a useful resource for identifying neuroanatomical differences and similarities between humans and other primate species and provide insight into the evolutionary variation and stasis of brain organization.

**Supplementary Information:**

The online version contains supplementary material available at 10.1007/s00429-023-02709-9.

## Introduction

Knowledge of the structure of lesser ape brains is critical to understanding the evolution of great ape brains, including humans. Comparative anatomy of rare species such as apes has greatly benefited from advances in structural neuroimaging, which now enables collection of high quality data from post-mortem fixed brains (Mars et al. 2014). Among others, diffusion MRI has enabled the production of white matter atlases of the brains of the macaque monkey (Schmahmann et al. 2007), squirrel monkey (Gao et al. 2016), and chimpanzee (Bryant et al. 2020). Although a few investigations into the morphometry and neural organization of the lesser apes have been undertaken (Apfelbach 1972; Schoenemann et al. 2005; de Sousa et al. 2010; Swiegers et al. 2019), thus far no study has examined the white matter tracts. Here, we provide a white matter atlas for the lar gibbon, a hylobatid which diverged from a common ancestor with humans approximately 16.8 million years ago (Carbone et al. 2014).

The atlas was constructed by identifying major white matter fiber tracts based on standardized anatomical landmarks that are directly comparable to previous studies in the human, chimpanzee, and macaque monkey brain (Mars et al. 2018b; Bryant et al. 2020). As in previous studies, these anatomical landmarks were used to create tractography ‘recipes’: a group of masks forming the seed, target, and exclusion regions of interest for a tractography algorithm to reconstruct the desired tract (Mars et al. 2018b; Warrington et al. 2020). The goal of these recipes is twofold. First, they are general enough to identify a tract of interest in every individual. Second, the masks are chosen such that the tractography is also selective to the tracts of interest. These protocols can be easily transformed to the space of individual scans in different datasets. It is the explicit goal of producing a library of tractography recipes that future modifications are easy to incorporate into the atlas and that interested researchers can easily test out alternative protocols to compare claims between rival definitions of a tract. A specialized tool, compatible with our data, is available for implementing these recipes (Warrington et al. 2020).

We employ these recipes to reconstruct a comprehensive set of 24 major white matter tracts in an *ex vivo* diffusion-weighted MRI dataset of a young adult (5.5 years) male lar gibbon brain using probabilistic tractography. We discuss the organization of major white matter fibers and their pattern of cortical terminations in the gibbon and compare them with our previously results in great apes and Old World monkeys. We observed that the gibbon temporal cortex shows hints of organizational principles previously identified as great ape specializations.

## Results

### Sulcal anatomy

We first reconstructed the cortical surface of the gibbon brain and performed sulcal labeling using the nomenclature of Petrides (2011) to provide a first reference frame for this brain. The gibbon sulcal and gyral morphology shares traits with both great apes (hominids) and Old World monkeys (OWM). Like the macaque, frontal cortex features a prominent principal sulcus running along the dorsolateral prefrontal cortex. Unlike in macaques or in humans, an arcuate sulcus or an inferior precentral sulcus is not present at the posterior limit of the PS. A segmented superior frontal sulcus (*sfs-a*, *sfsp*) is found in the dorsal prefrontal cortex. As in hominids, the posterior branch of the superior frontal sulcus joins the superior branch of the precentral sulcus. Also like hominids, orbital sulci (*los* and *mos*) are present, here visible along either side of the frontal operculum.

Moving posteriorly, gibbon parietal cortex has features that are intermediate between hominids and Old World monkeys. Like the macaque, the most prominent sulcus is a large intraparietal sulcus (*ips*) which originates posterior to the central sulcus, terminating orthogonal to the lunate sulcus. Notably, postcentral sulcus is not present. The caudal ramus of the superior temporal sulcus (*sts-a*) extends past the terminus of the lateral fissure and into inferior parietal cortex, nearly connecting with *ips*, similar to the macaque who lacks an angular or supramarginal gyrus separating the two sulci. In the gibbon, this *sts* extension is oriented vertically, unlike hominids or OWM, owing to the greater angle of the temporal lobe with respect to the parietal lobe. The prominent *sts* ramus (*sts-a*) is intermediate to the ascending ramus of the macaque *sts* and the complex observed branching of the *sts* in chimpanzees (Falk et al. 2018) and in humans (Ochiai et al. 2004).

Gibbon temporal cortex has a combination of OWM, hominid, and unique features. In addition to the *sts*, gibbon temporal lobe features an inferior occipital sulcus (*locs*) that reaches anteriorly past the posterior termini of the *sts* and its rami, further anterior than in humans (e.g., Malikovic et al. 2012) and more similar to macaques. The inferior temporal sulcus (*its*), previously suggested to be a hominid evolutionary innovation (Bryant and Preuss 2018), is visible in the lar gibbon as a small, shallow sulcus anterior to *locs*, suggesting this characteristic feature of great ape temporal lobe organization originated in the hominoid lineage. Like both OWM and hominids, the gibbon possesses an occipitotemporal sulcus (*lots*) in the ventral temporal lobe. An additional sulcus, the collateral sulcus (*cos*), runs medially to the lateral occipitotemporal sulcus in the gibbon (see Connolly 1950), which is also found in chimpanzees (Miller et al. 2020) and humans (e.g., Malikovic et al. 2012). In chimpanzees, the gyrus between these two sulci has been proposed to be homologous to the human fusiform gyrus and a possible hominoid innovation (Bryant and Preuss 2018).

**Fig. 1:**
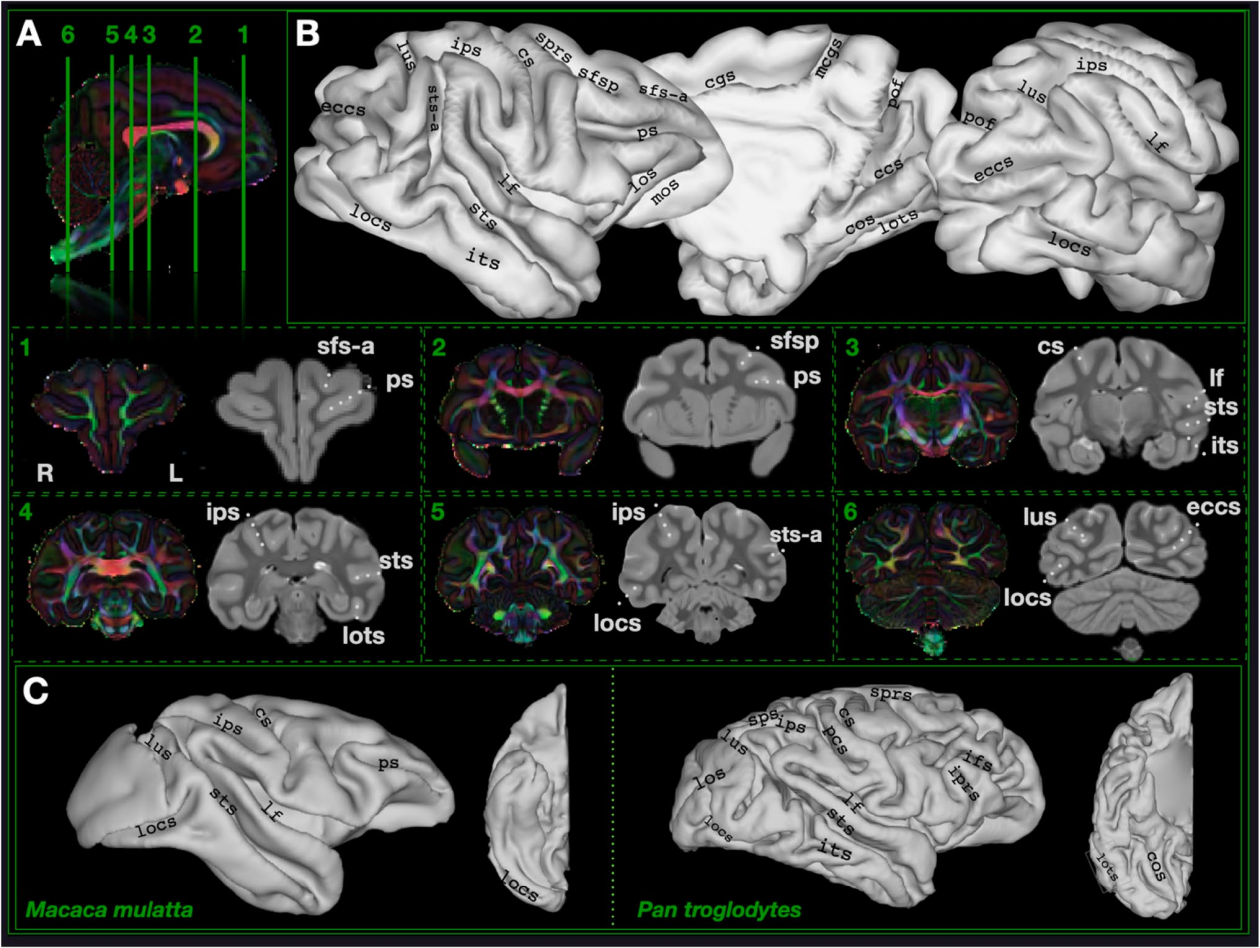
Sulcal boundaries in the lar gibbon with comparative anatomy in macaques and chimpanzees. **A** Coronal sections showing diffusion and grey matter at six locations throughout the brain. **B** Gibbon cortical surface reconstruction with sulcal labeling. **C** Macaque (three subject average from Roumazeilles et al. (2022)), gibbon, and chimpanzee (single subject from Roumazeilles et al. (2020)) for comparison. *ccs* calcarine sulcus, *cgs* cingulate sulcus, *mcgs* margin of the cingulate sulcus, *cos* collateral sulcus, *cs* central sulcus, *eccs* external calcarine sulcus, *ifs* inferior frontal sulcus, *iprs* inferior precentral sulcus, *ips* intraparietal sulcus, *its* inferior temporal sulcus, *lus* lunate sulcus, *lf* lateral fissure, *locs* lateral occipital sulcus, *lots* lateral occipitotemporal sulcus, *mos* medial orbital sulcus, *sprs* superior precentral sulcus, *pof* parieto-occipital fissure, *ps* principal sulcus, *sfs-a* superior frontal sulcus—anterior ramus, *sfsp* superior frontal sulcus—posterior ramus, *sts* superior temporal sulcus, *sts-a* superior temporal sulcus—ramus

Occipital cortex in the gibbon features a prominent lunate sulcus on the lateral surface, which is common to Old World anthropoids (although variable and often absent in humans (Malikovic et al. 2012)) and exhibits the characteristic primate calcarine sulcus medially. Unlike macaques, the gibbon occipital cortex features numerous sulci on the lateral surface, including a branched lateral calcarine sulcus (*eccs*) first identified by Connolly (1950). By contrast, chimpanzees and humans possess a lateral occipital sulcus separating the area into superior and inferior occipital gyri, while humans have an additional sulcus, the transverse occipital sulcus (Fig. 1).

### White matter tracts

**Fig. 2:**
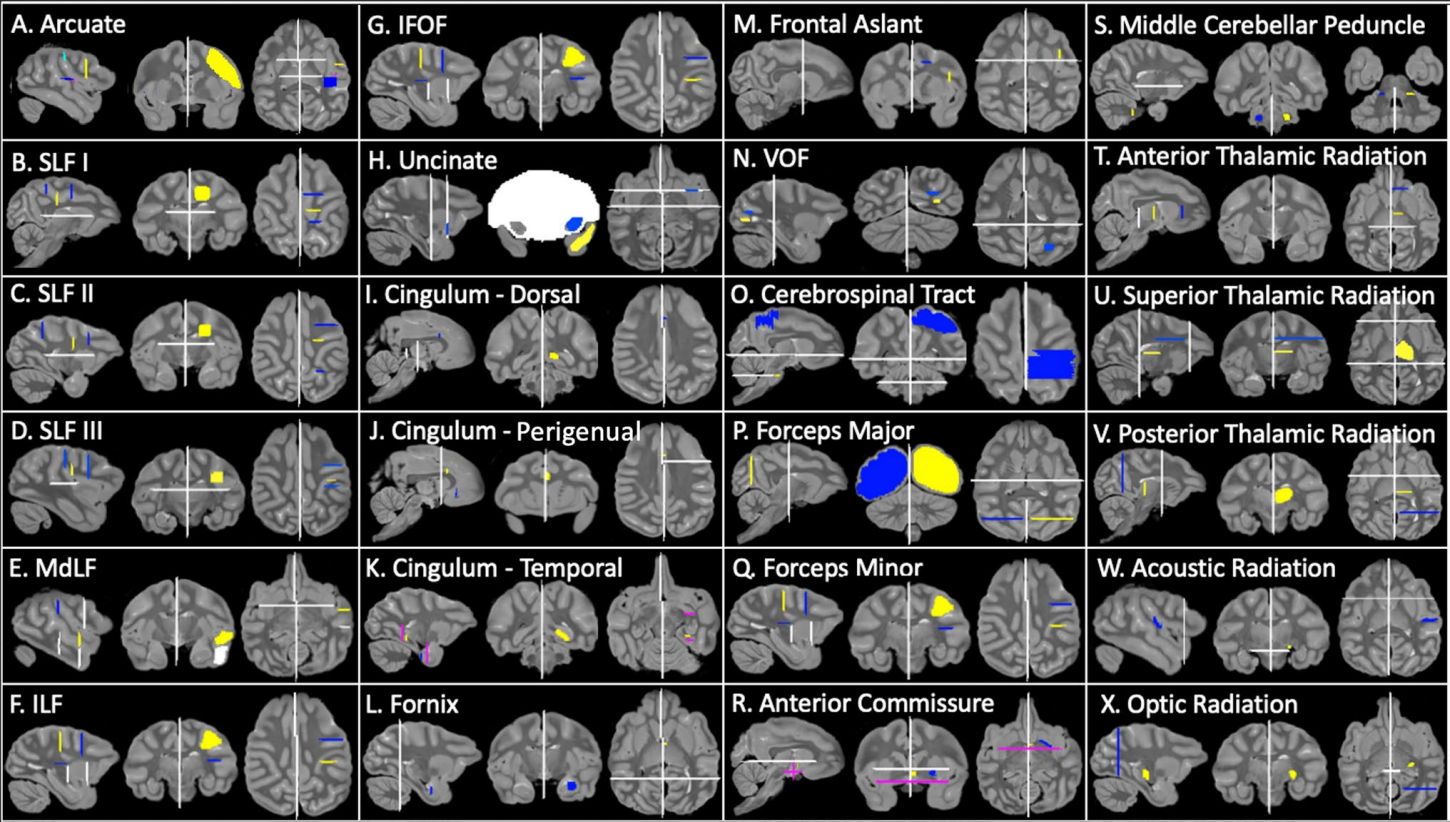
Lar gibbon tractography recipes. Seed ROIs (yellow), target ROIs (blue), exclusion masks (white), stop masks (fuchsia). Left hemisphere protocols are displayed. Arcuate protocol uses two targets: target 1 (light blue); target 2 (dark blue)

We created tractography recipes to reconstruct 24 tracts in the lar gibbon brain (Fig. 2). Our approach is to use anatomical landmarks to identify the bodies of tracts and subsequently guide tractography to reconstruct the tracts’ full path. Importantly, tractography allows the reconstruction of large white matter fiber bundles but does not necessarily respect synaptic boundaries. Thus, results obtained using this approach might best be compared to those obtained using blunt dissections, rather than tracer data. This approach of reconstructing tracts based on identification of the tract body has in the past proven robust (Thiebaut de Schotten et al. 2012; Mars et al. 2018a, b; Folloni et al. 2019), and does not suffer from the disadvantages commonly associated with tractography approaches that aim to mimic tracer data (Reveley et al. 2015; Donahue et al. 2016).

We discuss the course of the tracts below. Note that the tract protocols were defined to be as similar as possible to those previously described in other species (Mars et al. 2019; Bryant et al. 2020; Warrington et al. 2020), albeit adjusted for gibbon anatomy. We therefore aim to describe the results in the same terminology where possible, to allow the reader to form the best possible comparisons across species. In accordance with journal policy, we note this might give the appearance of text recycling.

For illustration purposes, we also provide surface projection maps of the major tracts (Fig. S1). To address the challenges of following fiber bundles into grey matter (Reveley et al. 2015), we employed a previously developed and validated approach in which the tract body is multiplied by the surface-to-white matter tractogram to create a connectivity blueprint (Mars et al. 2018b), illustrating the cortical territory of the tract on the cortical surface. We emphasize that the projection maps should not be interpreted as representations of synaptic connections with cortex but rather as depictions of broad patterns in fasciculo-cortical connectivity.

### Dorsal longitudinal tracts

The dorsal longitudinal fibers connecting the frontal lobe with the parietal and posterior temporal cortices are formed by the three branches of the superior longitudinal fascicle (SLF) and the arcuate fascicle. We here follow the convention of Schmahmann and Pandya (2006) of considering these as distinct tracts, even though the names have been used interchangeably in the literature. All four tracts have been identified using tractography in anthropoids (human: Makris et al. (2005), Rilling et al. (2008), Thiebaut de Schotten et al. (2012); chimpanzee: Bryant et al. (2020), Hecht et al. (2015); macaque: Schmahmann et al. (2007)).

**Fig. 3:**
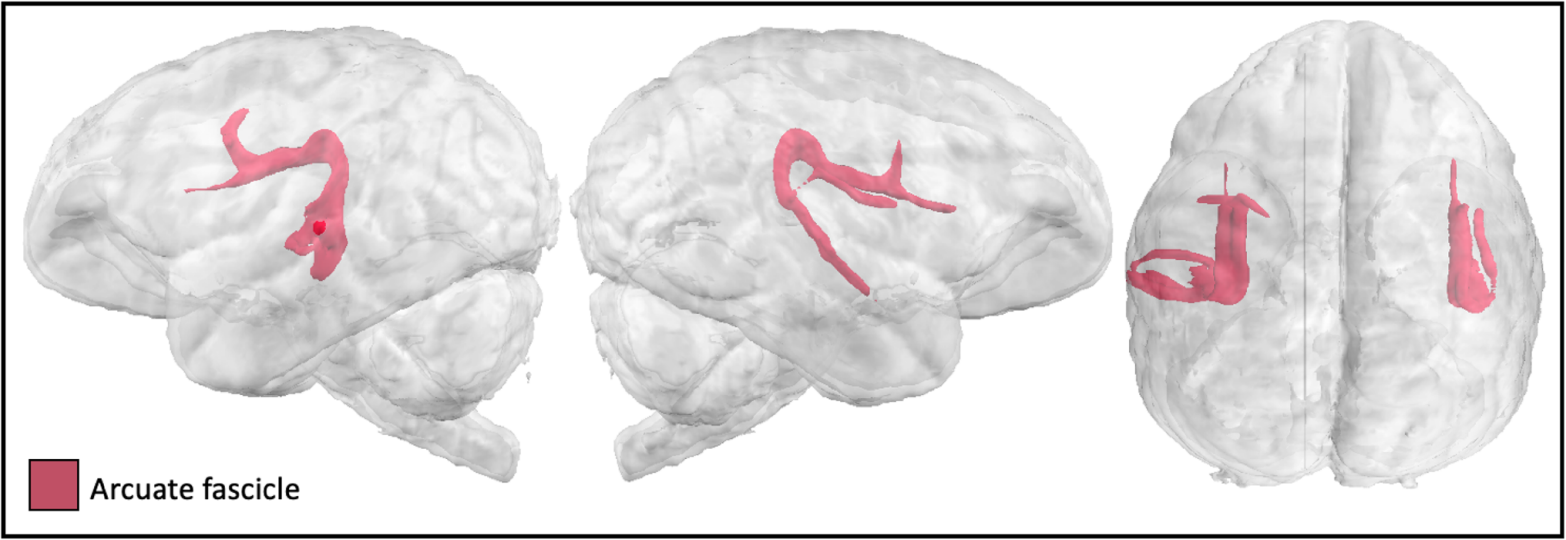
Arcuate fascicle in the lar gibbon

The gibbon arcuate tractogram extended between the superior temporal gyri and the ventral prefrontal cortices (Fig. 3, S2). Surface projections showed anterior projections reaching ventral prefrontal areas and posterior projections reaching posterior to the middle part of the superior temporal gyrus and *sts* (Fig S1a).

**Fig. 4:**
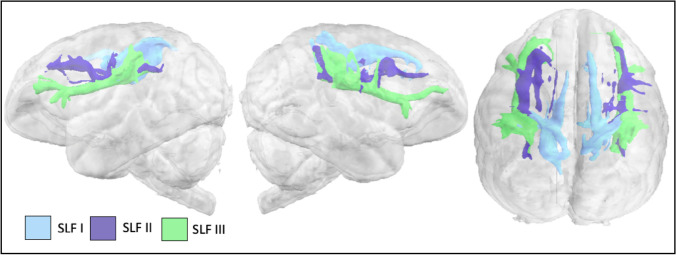
SLFs I, II, and III in the lar gibbon

Superior longitudinal fascicle I (SLF I) extended from the superior parietal lobule to the dorsal prefrontal cortex superior to the *sfs-a* (Fig. 4). Superior longitudinal fascicle III (SLF III) reached parietal areas inferior to *ips* and ventral prefrontal cortex inferior to *ps*. The second superior longitudinal fascicle (SLF II) ran roughly equidistant between SLF I and SLF III, reaching lateral prefrontal cortices between *ps* and *sfs-a* with posterior parietal areas inferior to *ips*. Surface projections indicate that SLF II and SLF III have more expansive ventral prefrontal projections and inferior parietal projections, while SLF I streamlines are focused in dorsal prefrontal and superior parietal areas (Fig S1b–c).

### Temporal association tracts

**Fig. 5:**
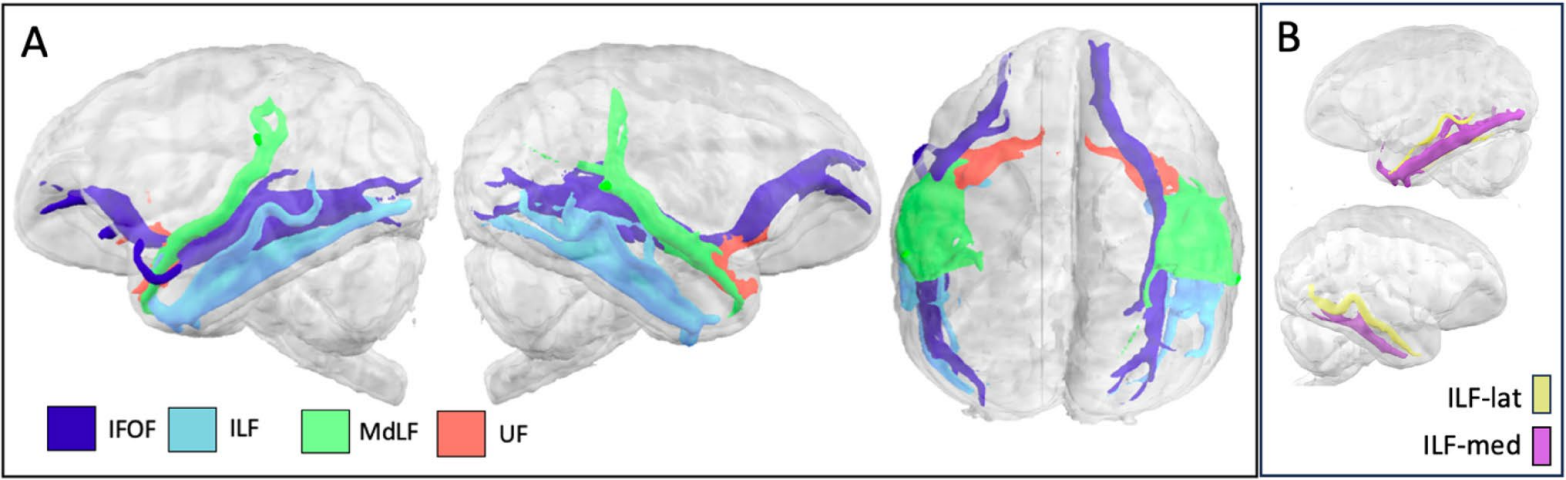
**A** Major fasciculi that course through the temporal lobe: (IFOF (inferior fronto-occipital fascicle), ILF (inferior longitudinal fascicle), MdLF (middle longitudinal fascicle) and UF (uncinate fascicle) in the lar gibbon. **B** Lateral (ILF-lat) and medial (ILF-med) subdivisions of the ILF in the gibbon

The middle longitudinal fascicle (MdLF) in lar gibbon spanned the length of superior temporal gyrus (STG), extending from parietal cortex inferior to *ips* and anterior to *pof* towards the temporopolar region of anterior STG (Fig. 5). Surface projections are concentrated in middle and posterior STG as well as *sts* and inferior parietal cortex (Fig S1e).

The inferior fronto-occipital fasciculus (IFOF) in gibbon extended from the occipital lobe, through the temporal lobe, medial to the ILF and MdLF, into the prefrontal cortex via the extreme/external capsule, coursing superiorly to the uncinate fascicle to the prefrontal cortex, where it splits into terminations inferior and to a lesser extent superior to the *ps* (Fig. 5A). These patterns are also discernible in surface projections, with streamlines concentrated along *sts* and ventral frontal cortex (Fig. S1f).

The inferior longitudinal fasciculus (ILF) coursed through the ITG and MTG, spanning temporopolar regions to posterior MTG and extending to inferior lateral occipital cortex (Fig. 5A). Streamlines from gibbon ILF to the cortical surface encompassed the length of ITG as well as occipital and inferior parietal areas (Fig. S1g). Previous work in humans has suggested that the ILF in that species can be dissociated into subcomponents (Latini et al. 2017) and previous comparative work shows this could be demonstrated in great apes, but not macaques (Roumazeilles et al. 2020). To test where gibbon ILF falls in this spectrum, we adapted the recipes for lateral and medial branches of the ILF developed for great apes. This showed that in the gibbon, ILF has a clear medial branch which reaches inferior to *its* and a smaller lateral branch that extends superior to *its* (Figs. 5B, S3).

The uncinate fascicle (UF) extended from the temporopolar region of the STG to inferior prefrontal cortex, passing through the extreme/external capsule in close apposition and just inferior to the IFOF, between the *los* and *mos* (Fig. 5A). Surface projections were concentrated in anterior STG and orbital and ventral prefrontal areas surrounding the anterior limit of the *ps* (Fig. S1h).

### Limbic tracts

**Fig. 6:**
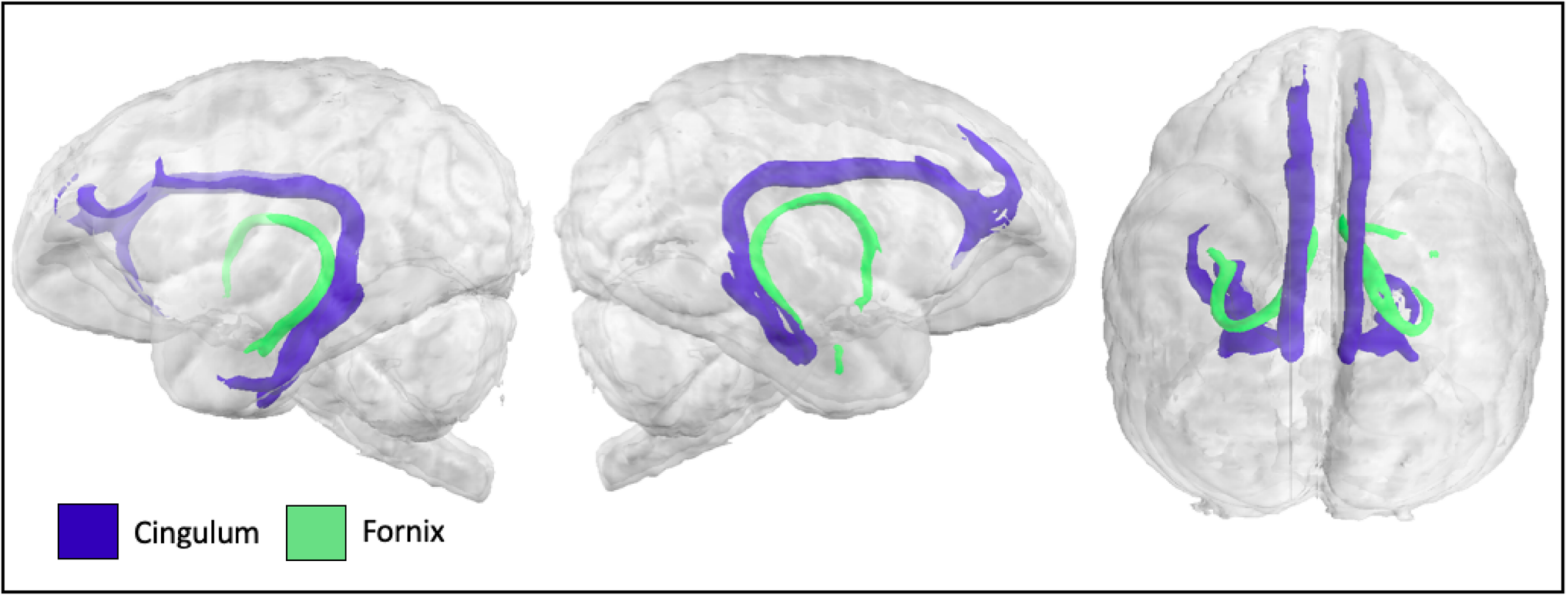
Cingulum bundle and fornix in the lar gibbon

The cingulum bundle was reconstructed by combining the results from three segments—temporal, dorsal, and peri-genual–extending from the parahippocampal gyrus, through the medial posterior temporal lobe, coursing rostrocaudally superior to the corpus callosum, and terminating in medial prefrontal cortex (Fig. 6). Dorsal prefrontal projections medial to *sfs-a*, and ventral prefrontal projections medial to *los* were discernible. The fornix extended from the medial temporal lobe just superior to the cingulum to the mammillary bodies and hypothalamus (Fig. 6).

### Short tracts

**Fig. 7:**
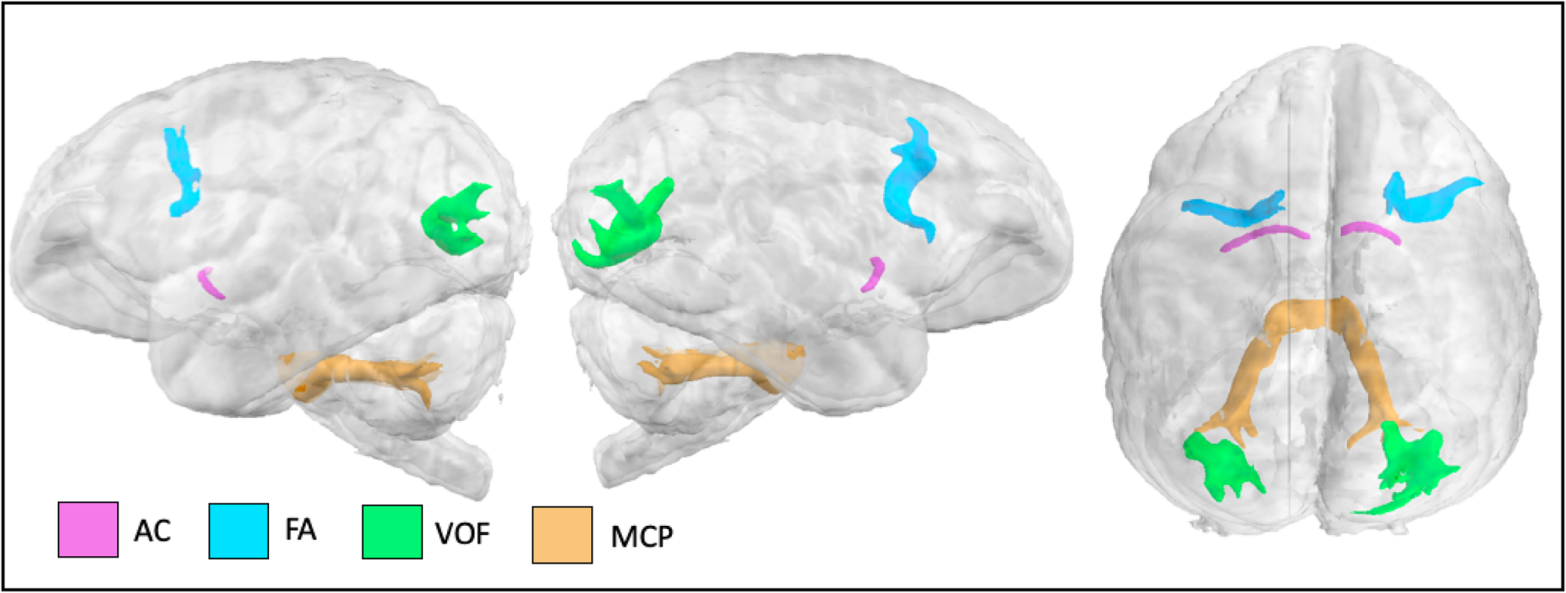
AC (anterior commissure), FA (frontal aslant), VOF (vertical occipital fasciculus), and MCP (middle cerebellar peduncle) in the lar gibbon

The frontal aslant (FA) connects ventrolateral prefrontal cortex with dorsal frontal cortex. In the gibbon, this tract is rather thin and arches around the principal sulcus (Fig. 7).

The vertical occipital fasciculus (VOF) connects dorsal and ventral surfaces of the occipital lobe. In the gibbon, this bundle arches between these territories, medial to the lunate sulcus (Fig. 7).

### Interhemispheric tracts

The anterior commissure connects ventral and anterior temporal cortices of both hemispheres, including the amygdalae. (Fig. 7). The middle cerebellar peduncle is a collection of fiber tracts that arises in the pontine nuclei and project to the opposite cerebellar hemisphere (Fig. 7).

### Corticospinal and somatosensory pathways

**Fig. 8:**
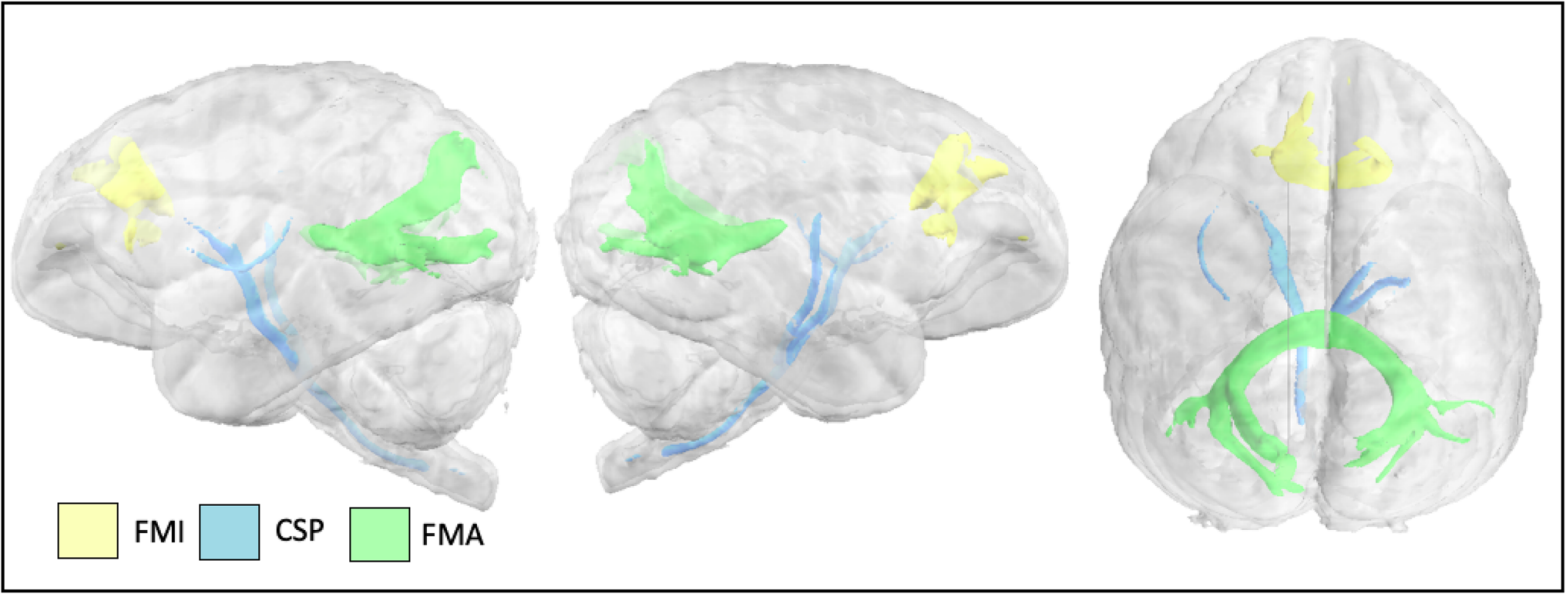
Corticospinal and somatosensory pathways (CSP), forceps major (FMA) and minor (FMI) in the lar gibbon

The corticospinal and somatosensory pathways (CSP) send projections from the motor and somatosensory cortices to the spinal cord. Streamlines from the CSP tractogram in the gibbon stretched toward precentral and postcentral gyri (Fig. 8).

The forceps major and minor are components of the corpus callosum (passing through the splenium and the genu, respectively). Forceps major tractograms in the gibbon showed both superior and inferior branching as they reached the occipital lobe (Fig. 8); forceps minor streamlines reached dorsal frontal cortex medial to the *sfs*.

### Thalamic projections

**Fig. 9:**
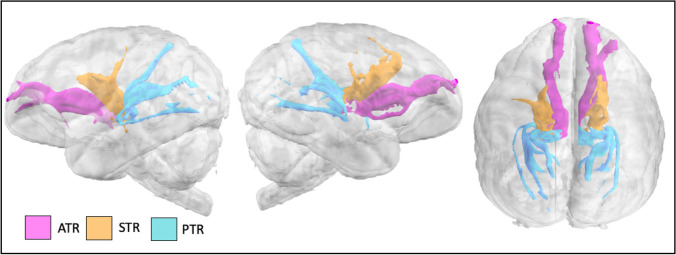
Anterior, superior, and posterior thalamic radiations (ATR, STR, and PTR) in the lar gibbon

Anterior, superior, and posterior thalamic radiations project from the thalamus to frontopolar, dorsal frontal cortex anterior to *cs*, and occipital/parietal cortices, respectively (Fig. 9).

**Fig. 10:**
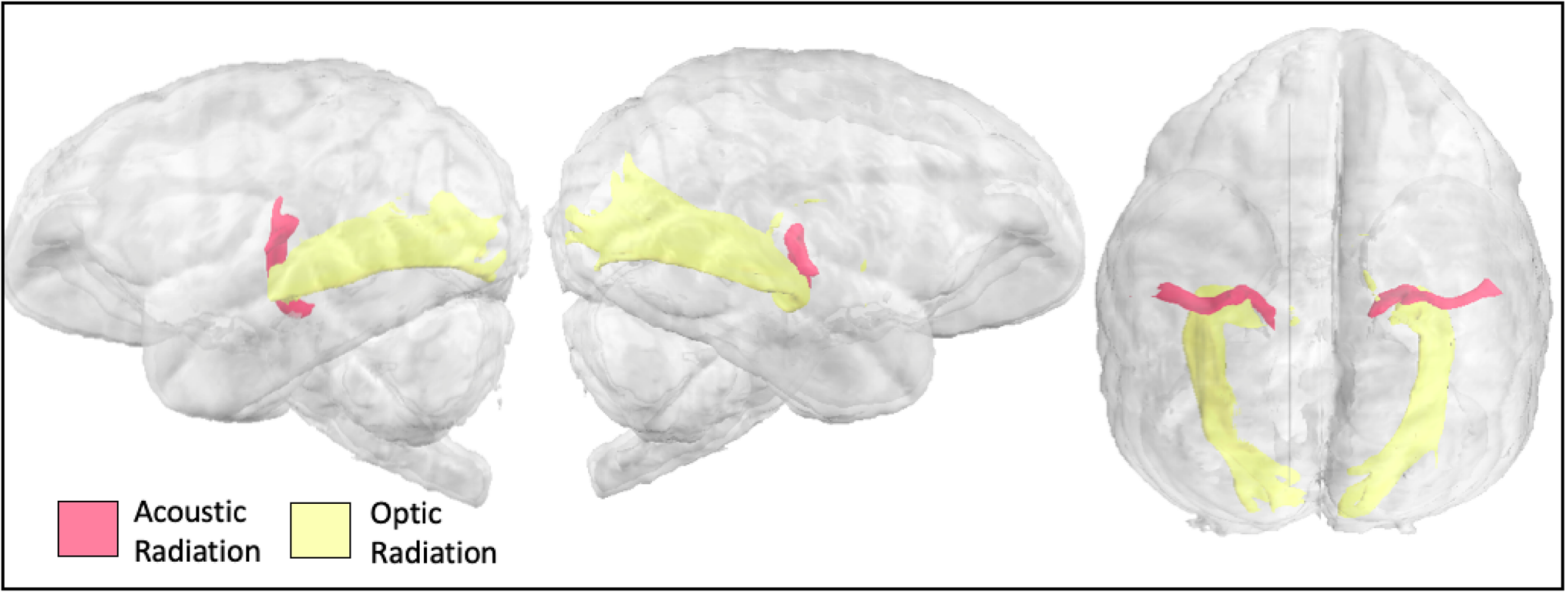
Optic and acoustic radiations in the lar gibbon

Optic and acoustic radiations connect the small geniculate nuclei (middle geniculate nuclei for acoustic; lateral for optic) to the primary sensory cortices. Gibbon optic and acoustic radiation tractograms showed expected projections toward occipital cortex (focused in the occipital pole) and planum temporale, respectively (Fig. 10).

## Discussion

**Fig. 11:**
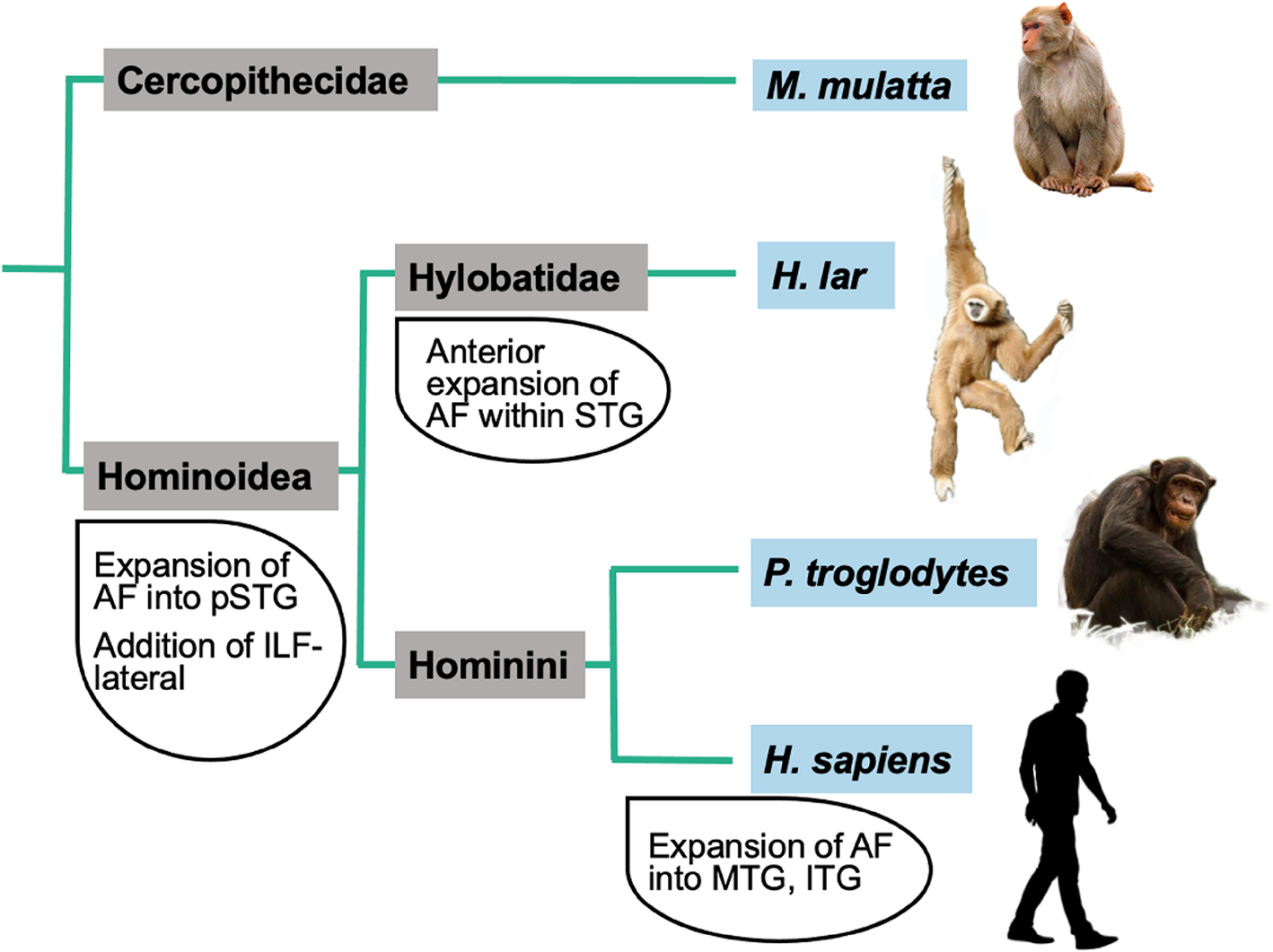
Summary of findings in evolutionary context

We have presented a library of recipes to reconstruct the major white matter tracts in the gibbon brain. Similar libraries are available in the human, chimpanzee, and macaque (Mars et al. 2018b; Bryant et al. 2020; Warrington et al. 2020). Creating such a library for the gibbon, a hylobatid, allows direct quantitative investigations on general principles of primate brain organization as well as on specializations of hominoids and hominids (Fig. 11).

The use of diffusion MRI tractography to characterize the white matter anatomy of less well-studied species like the lar gibbon offers several advantages. The non-destructive and non-invasive nature of MRI makes it well-suited for a number of rarer species (Mars et al. 2014) and permits directly comparable analyses of brain connectivity across species (Mars et al. 2018b). Practical and ethical reasons prohibit the use of traditional techniques such as tract tracing in many primate species. Post-mortem diffusion MRI data can be stored and shared via data banks such as the Digital Brain Bank (open.win.ox.ac.uk/DigitalBrainBank; Tendler et al. (2022)). The gibbon library produced here is compatible with the XTRACT tool of the FSL software (Warrington et al. 2020). Creating such a standardized set of recipes facilitates the use of common terminology, offers the chance for others to build upon and improve our protocols, and will aid in solving disputes of tract identifications.

In the next sections, we will discuss how the tracts identified by our recipes can enrich our understanding of white matter evolution in humans and apes and explore some possible functional implications for the observed similarities and differences; however, in-depth quantitative investigations comparing white matter architecture of the gibbon with that of other species will be reserved for future communications.

Our findings indicate that the temporal connections of the gibbon arcuate fasciculus reach further anteriorly in STG than in macaques, and include connections into *sts*, similar to chimpanzees (Fig. 3). Interestingly, the gibbon arcuate fasciculus reaches even further anteriorly in STG than has previously been described in chimpanzees both in averaged results (Bryant et al. 2020) and on an individual level (Fig. S2). The most parsimonious explanation is that temporal expansions to the arcuate fasciculus were present in the last common ancestor to hominoids approximately 16 million years ago and were further modified in the great ape and human lineages. Alternatively, it could suggest a convergent evolution of the arcuate fasciculus in two of the many extant and extinct hominoid species, lar gibbons and humans. In humans, the arcuate fasciculus is critical to language processing, and the ability of chimpanzees and bonobos to acquire some semantic comprehension and manual language production (Gardner and Gardner 1969; Fouts 1973; Savage-Rumbaugh et al. 1986) suggests that the more extensive STG connectivity of the arcuate fasciculus in these species compared with macaques may facilitate these abilities. Experimental language studies have not been carried out in gibbons, but analysis of wild gibbons’ calls indicates the presence of precursors to syntax and semantic processing; the former in the form of combinatorial hierarchical structuring (Inoue et al. 2017) and the latter in the form of referential signaling (Clarke et al. 2006). The human-unique expansion of arcuate fasciculus connectivity in humans to include middle and inferior portions of the temporal lobe is likely related to the human capacity for semantic richness and syntactic complexity. The structure of the gibbon arcuate fasciculus described here suggests the initial expansions of this fasciculus, a required foundation for human language, originated in hominoid expansions of the arcuate fasciculus into STG and *sts*.

Dorsal longitudinal tracts belonging to the SLF were identified running between the parietal and frontal cortex. In most anthropoid primates to date, the SLF is subdivided into three distinct branches that form parallel pathways between the parietal and frontal cortex (Thiebaut de Schotten et al. 2011). We see a similar organization in the gibbon: the parietal cortex in the gibbon is quite convoluted, with the uprising branch of the STS into the inferior parietal lobule particularly noticeable. Terminations of the SLF were detected both anteriorly and posteriorly of this branch, indicating it is not likely to be a demarcation of parietal cortex. Rather, the lunate sulcus seems to be a better landmark for this; in gibbons it is rotated such that it is oriented more vertically than in chimpanzees or macaques.

The ILF is a visual association pathway connecting occipital regions with ventral temporal association areas. Previously, a hominid-unique component, ILF-lateral, was identified (Roumazeilles et al. 2020), following earlier dissection work in the human (Latini et al. 2017). ILF-medial, found in macaques as well as great apes, runs along the ITG, but ILF-lateral expands laterally into the white matter of MTG in hominids. Here, we find that gibbons have an ILF organization reminiscent of that of the great apes, suggesting ILF-lateral expansion occurred in the hominoid lineage (Fig. 5B). Reconstruction of ILF-medial and ILF-lateral in gibbon, alongside humans and chimpanzees, further supported the interpretation that gibbon ILF, like hominids, comprise dissociable subcomponents (Fig. S3) Combined with the presence of the *cos*, this suggests hominoids possess a series of temporal lobe modifications for visual object processing unique to the lineage.

Finally, we observed that the forceps major in gibbon branches into distinct dorsal and ventral occipital components (Fig. 8), unlike humans and to a greater degree than observed in macaques and chimpanzees (Fig. S2, Bryant et al. 2020). Given the unique angle of parietal to temporal cortex observed in gibbons, this gross morphological difference may be due to vertical lengthening of occipital cortex in gibbons, and when compared to humans, possibly compounded by the compression of these branches by parietal expansion occurring in the human lineage (Bruner 2018).

Our gibbon dataset was based on a single, relatively young, male individual. Our results should therefore be read as preliminary and awaiting confirmation in a larger sample. However, given the importance of adding data from the lesser apes to the emerging picture of primate connectivity, we felt it important to communicate these results. These results can be added to the increasing library of white matter maps of the primate brain. Apart from the human, chimpanzee, and macaque results referred to earlier, these also include partial atlases in other great apes, Old and New World monkeys, and prosimians (Roumazeilles et al. 2020, 2022; Bryant et al. 2021).

Diffusion MRI tractography has several advantages for comparative neuroscience research but has some important differences when directly compared to more traditional neuroscientific methods. Studies comparing tract tracing with diffusion tractography in ex vivo macaques have found comparable results (Jbabdi et al. 2013; Azadbakht et al. 2015; van den Heuvel et al. 2015; Donahue et al. 2016), and high angular resolution approaches have successfully been used on difficult-to-reconstruct tracts such as the acoustic radiation (Berman et al. 2013). Tractography can produce false positives, and the best approach to mitigate this is the use of strong anatomical priors (Maier-Hein et al. 2017). As in previous studies (Bryant et al. 2020), we use standardized protocols based on strong anatomical knowledge from other species, including those for which a wealth of tracer data is available. To guard against false negatives, these data can be compared with results from data-driven methods; for example, in chimpanzees, anatomically-driven and data-driven approaches produce comparable results (Mars et al. 2019).

Connectivity is of course but one aspect of brain organization on which to compare different species. It has proven a useful measure, as connectivity is generally considered to be important for function and can be compared reliably across species (Mars et al. 2018a, b). Another modality that has been employed in recent large-scale comparative studies is that of cortical folding patterns. Within primates, brain size predicted gyrification patterns better than evolutionary lineage (Heuer et al. 2019). Although cortical folding does not speak to the internal organization of the brain, a number of comparative studies have suggested cortical folds are a reliable metric to chart brain evolution (Amiez et al. 2019; Miller and Weiner 2022). Ultimately, comparative neuroscience will benefit from direct comparisons across levels of brain organization, as was recently done in a study comparing tissue properties and connectivity in the primate temporal lobe (Eichert et al. 2020).

In summary, our results suggest that gibbon white matter tracts share features with chimpanzees and humans not found in rhesus macaques. Specifically, the gibbon brain demonstrates expansions of the AF into the STG and *sts*, and the ILF into lateral temporal cortex, unlike what has previously been observed in cercopithecoid monkeys. Similar modifications to the AF and ILF have been observed in great apes (Rilling et al. 2008; Bryant et al. 2020; Roumazeilles et al. 2020), suggesting that these fascicular specializations appeared over 16 mya, prior to divergences within the hominoid lineage.

This gibbon white matter atlas is presented as a resource and a starting point for future quantitative and comparative analyses. Future work will take advantage of this atlas and accompanying tract recipes to reconstruct tracts in hylobatid species and populations, to use as a reference to create tractography protocols for other primate species, and to probe in more detail the neuroanatomical adaptations unique to humans and great apes.

## Materials and methods

### Data

Scan protocols were similar to those in our previous communications for post-mortem small primate brains (Bryant et al. 2021; Roumazeilles et al. 2022). Diffusion MRI scans of a male lar gibbon (*Hylobates lar*; *n* = 1, body mass: 5.5 kg, brain mass: 112 g, 5.5 years old) were acquired in a were acquired locally on a 7 T magnet with an Agilent DirectDrive console (Agilent Technologies, Santa Clara, CA, USA) using a 2D diffusion-weighted spin-echo protocol with single line readout (DW-SEMS, TE/TR: 25 ms/10 s; Matrix = 128 × 128; number of slices: 128; resolution: 0.6 × 0.6 × 0.6 mm; diffusion data were acquired over the course of ~ 52.5 h). Eight non-diffusion-weighted (*b* = 0 s/mm2) and one hundred twenty-eight diffusion-weighted (*b* = 4000 s/mm2) volumes were acquired with diffusion directions distributed over the whole sphere. The brain was soaked in PBS before scanning and placed in fluorinert during the scan. The brain was obtained from Copenhagen Zoo, Denmark, where the animal was euthanized for management reasons, unrelated to this study (Bertelsen 2018). Following euthanasia, the carotid arteries were cannulated, and the head was perfused with an initial rinse of 0.9% saline solution at a temperature of 4 ºC followed by 4% paraformaldehyde in 0.1 M phosphate buffer (PB) at 4 ºC. The brain was removed from the skull and post-fixed in 4% paraformaldehyde in 0.1 M PB (24 h at 4 ºC).

Scan data were converted to NIFTI format, reoriented to approximate AC/PC orientation with the origin set at the middle of the anterior commissure, and preprocessed using tools from FSL (www.fmrib.ox.ac.uk/fsl). These steps are implemented in the ‘phoenix’ module of the MR Comparative Anatomy Toolbox (Mr Cat; www.neuroecologylab.org). We used bedpostX to fit a crossing fiber model to each dataset (Behrens et al. 2007), allowing up to three fiber orientations per voxel. This tool produces voxel-wise posterior distributions of fiber orientations that are subsequently used for probabilistic tractography.

### Surface reconstruction

The gibbon cortical surface was reconstructed from the structural scans using a customized pipeline based on FreeSurfer v6.0 (Fischl 2012). Processing steps included tissue segmentation of grey and white matter, identification of subcortical structures, and surface reconstruction. To obtain successful reconstructions of the non-human primate brains, the FreeSurfer pipeline was complemented with tools from FSL v6.0.1 (Jenkinson et al. 2012), ANTs (Avants et al. 2011), and MATLAB (2017a, The Mathworks, Inc., Natick, Massachusetts, United States).

Diffusion images containing samples from the distribution on anisotropic volume fraction (mean_f1samples in FSL terminology) were adjusted to obtain a T1-like contrast with low voxel intensities in gray matter voxels and high intensities in white matter voxels. Tissue segmentation was performed using FreeSurfer with manual corrections where necessary to correct segmentations of white matter, cerebellum, and subcortical structures. Surfaces were converted to GIFTI format and a mid-thickness surface was created by averaging the pial and white matter border surfaces. For display purposes, the mid-thickness surface was smoothed (strength 0.5, 15 iterations) using the Workbench Command tools of Connectome Workbench (Marcus et al. 2011).

### Tractography

Probabilistic diffusion tractography was performed using FSL’s probtrackx2 (Behrens et al. 2007). Recipes consisting of seed, waypoint (target), exclusion, and/or stop masks for each white matter tract of interest were drawn in volume space of the gibbon following the approach previously established for the human and macaque (Mars et al. 2018a, b; Warrington et al. 2020). Details of these protocols follow in the next section. Tractography was run in two directions (seed to target and target to seed) with the following parameters: each seed voxel was sampled 10,000 times, with a curvature threshold of 0.3, step length of 0.5 mm, and a number of steps of 3200. The resulting tractograms were normalized by dividing by the waypoint number and thresholded at 0.0005. Results were visualized with Matlab (MATLAB and Statistics Toolbox Release 2012b, The MathWorks, Inc., Natick, Massachusetts, United States).

### Tractography recipes

Protocols are described below and are defined as similarly as possible as those previously defined for other primates (Mars et al. 2018b; Bryant et al. 2020; Warrington et al. 2020), while adjusted for gibbon anatomy. In accordance with journal policy, we therefore note there might be some recycling of text of those previous publications were appropriate. Unless noted otherwise, all protocols included a bilateral exclusion mask consisting of a sagittal section isolating tracts within the two hemispheres with the exception of the middle cerebellar peduncle and anterior commissure.

### Dorsal tracts

#### Superior longitudinal fasciculi and arcuate fasciculus

The arcuate fasciculus (AF) was reconstructed with a one seed, two target “wayorder” approach, in which target ROIs were specified in a particular order (seed to target 1, then target 1 to target 2). Here, a large seed was drawn in coronal section just posterior to the posterior terminus of the ps and extending from the frontal operculum to the los, while target 1 was placed in coronal section superior to the posterior limit of the *lf *and target 2 in horizontal section in the white matter of the superior temporal gyrus (STG; Fig. 2A). A stop mask was drawn in the area encompassing the auditory core to avoid contamination from the acoustic radiation (Fig. 2A).

Superior longitudinal fasciculi (SLFs) were reconstructed using a one seed, two target approach, in which the seed ROI was drawn in the center of the tract and a target ROI was drawn at the anterior and posterior ends of the tract (Figs. 2B-D). For SLF I, the seed was drawn slightly anterior to the central sulcus, in the white matter inferior to *sprs*, with one target in the medial to the *sprs* and a posterior target, the white matter of parietal cortex medial to the dorsal limit of *ips* (Fig. 1B). SLF II involved a seed mask anterior and deep to the central sulcus, with target masks in the white matter deep to and between the *sfsp* and *ps* and inferior and posterior to the *ips*, between *sts-a* and *lus* (Fig. 2C). For SLF III, the seed was placed in the white matter posterior the *cs*, with a target in the white matter inferior to the *ps* and superior to the posterior terminus of the *lf* (Fig. 2D). Dorsal tract protocols employed exclusion masks that excluded the basal ganglia (Fig. 2B).

### Temporal tracts

#### Middle longitudinal fasciculus

MdLF was reconstructed using a seed in STG, slightly anterior to the central sulcus, and a target also in the STG, just anterior to the posterior terminus of the Sylvian fissure (Fig. 2E). Exclusion masks were inferior to *sts* and in the prefrontal cortex (Fig. 2E, to prevent contamination with ILF and IFOF.

#### Inferior longitudinal fasciculus

The seed mask was located in a coronal section below *sts* just posterior to the temporal pole, and the target mask was located in a coronal section inferior to *sts* and superior, medial, and inferior to the anterior end of the locs (Fig. 2F). Exclusion masks were placed in coronal sections of the STG (Fig. 2F.

#### Inferior fronto-occipital fasciculus

IFOF recipes consisted of a large coronal slice in the occipital lobe at the level of the *lus* for the seed and a coronal slice in the prefrontal cortex, just anterior to the genu of the corpus callosum, for the target (Fig. 2G). To restrict streamlines to those that pass through the extreme/external capsule, a large exclusion mask was drawn as a coronal slice with two lacunae at the extreme/external capsule (Fig. 2G).

#### Uncinate fasciculus

Like the IFOF protocol, the uncinate protocol also used a large coronal exclusion mask with lacunae at the extreme/external capsule. The seed was placed in the white matter close to the temporal pole and the target was drawn in the extreme/external capsule (Fig. 2H). A second exclusion mask was placed posterior to the basal ganglia to prevent contamination from longitudinal fibers from the IFOF.

### Limbic tracts

#### Cingulum bundle

This tract was divided into three separate protocols—cingulum dorsal (CBD), cingulum peri-genual (CBP), and cingulum temporal (CBT). The seed and target for CBD were placed in the cingulate gyrus, at the level of the precuneus and the dorsal part of the genu, respectively (Fig. 2I). The CBP seed mask was placed dorsal to the genu and the target at the ventral terminal point of the genu (Fig. 2I). The CBT recipe involved a seed in the posterior parahippocampal gyrus and one in the anterior parahippocampal gyrus, and two stop masks to avoid picking up occipitotemporal and extreme/external capsule fibers (Fig. 2K).

#### Fornix

The fornix protocol included a seed in the mammillary bodies and a target in the hippocampal formation (Fig. 2L). In addition to the bilateral exclusion mask, a coronal section of occipital lobe was excluded to avoid contamination from posterior fiber bundles (Fig. 2L).

### Short tracts

#### Frontal aslant

A seed was placed in a parasagittal section at the white matter stem between *ps* and *los*, and the seed in an axial section of white matter medial to SFS (Fig. 2M). The exclusion mask included a coronal slice just posterior to the seed and target masks, to avoid including streamlines from dorsal fasciculi (Fig. 2M).

#### Vertical occipital fasciculus

The vertical occipital fasciculus (VOF) recipe contains a seed and a target in the white matter of the occipital lobe, medial to the eccs, the seed superior and the target inferior to the fundus of lus (Fig. 2N). An exclusion mask consisting of a coronal section at the level of the posterior temporal lobe was used to avoid the inclusion of longitudinal tracts (Fig. 2N).

### Corticospinal and somatosensory pathways (CSP)

A seed was placed in the central medial portion of the pons, with a target encompassing the superior parietal lobule, inferior parietal lobule, and postcentral gyrus (Fig. 2O). Exclusion masks were placed in an axial section at the level of the midbrain, which excluded streamlines outside of the midbrain/brainstem (Fig. 2O).

### Interhemispheric tracts

#### Forceps major and minor

A seed mask consisting of a coronal slice through one occipital lobe, a target mask consisting of a coronal slice through the other occipital lobe, a coronal exclusion mask at approximately the level of the central sulcus, and a sagittal exclusion mask between the occipital lobes were used to reconstruct the forceps major (Fig. 2P). The forceps minor recipe was similar, with target and seed masks in coronal sections of the prefrontal cortex (Fig. 2Q).

#### Anterior commissure

A seed was placed at the midline of the anterior commissure and a target in the white matter between the globus pallidus and the putamen, based on descriptions from Dejerine and Dejerine-Klumpke (1895). An exclusion mask was placed superior to the seed and target masks and stop masks were placed posterior to the seed and target masks, to prevent streamline contamination from the rest of the basal ganglia, and in an axial section at the level of the extreme/external capsule, to prevent streamlines from going into the ventral pathway (Fig. 2R).

#### Middle cerebellar peduncle

A seed was placed in the white matter stem of one cerebellar hemisphere and a target in the opposite (Fig. 2S). An exclusion mask was placed sagittally between the two cerebellar hemispheres and axially at the base of the thalamus. (Fig. 2S).

### Thalamic projections

#### Thalamic radiations

Anterior thalamic radiation recipes consisted of an axial seed in the anterior third of the thalamus (Wakana et al. 2007), with a target in the white matter of the prefrontal cortex just anterior to the basal ganglia, in the anterior thalamic peduncle (Fig. 2T). An exclusion mask was placed posterior to the thalamus. The superior thalamic radiation protocol consisted of a seed in the superior half of the thalamus, and a target in the precentral and postcentral gyri. Exclusion masks were placed coronally, posterior to the thalamus and anterior to the precentral gyrus (Fig. 2U). To reconstruct the posterior thalamic radiation, a seed in the posterior thalamus and a coronal mask in the occipital lobe were used (Fig. 2V). Exclusion masks were placed anterior and inferior to the thalamus (Fig. 2V).

#### Acoustic and optic radiations

For the acoustic and optic radiations, seeds were placed in the medial geniculate nucleus and lateral geniculate nucleus of the thalamus, respectively. A target was placed in the planum temporale for the acoustic radiation (Fig. 2W) and as a coronal section at approximately the level of the lunate sulcus for the optic radiation (Fig. 2X).

### Surface projection maps

Cortical surface representations of each tract were created to establish which cortical territories are reached. Since there are known issues of gyal bias and superficial white matter (Reveley et al. 2015; Schilling et al. 2017), when tracking toward the cortical grey matter, we employed a recent approach that aims to address some of these issues by multiplying the tractography results with a whole-brain connectivity matrix (Mars et al. 2018b; Eichert et al. 2019).

The above-described tractograms for each tract were normalized and thresholded, resulting in a (tract) x (brain) matrix for each tract. This tractogram matrix was then multiplied by a whole-brain (surface) x (brain) connectivity matrix derived from seeding in the mid-thickness surface and tracking to the rest of the brain. The whole-brain connectivity matrix was based on a surface reconstruction of the gibbon brain (5 k vertices).

### Comparative analyses

For analysis purposes, previously developed tractography recipes for AF, FMA, ILF, and CST were used to reconstruct these tracts in humans, chimpanzees, and macaques. These data comprised 32 humans from the Human Connectome Project dataset, 29 chimpanzees from a previously acquired dataset from the Emory National Primate Research Center, and 6 ex vivo rhesus macaques scanned at the University of Oxford (for further information on sample acquisition and scanning protocols, see Bryant et al. 2020). Tractography parameters were identical to the gibbon except for a slightly more conservative curvature threshold (0.2) which has been validated in previous studies of humans and non-human primates (e.g., Warrington et al. 2020).

To explore the anatomy of subcomponents of the ILF (ILF-lat and ILF-med, previously described in Roumazeilles et al. 2020), tractography recipes were developed for these ILF segments using anatomical references from the clustering results generated in humans, chimpanzees, and macaques (Roumazeilles et al. 2020). ILF-lat and ILF-med recipes were then adapted for gibbon.

Tractography for gibbon was carried out as described above. In the three non-gibbon primates, individual tractograms were thresholded, binarized, and summed, and then visualized with FSLeyes (FSL v6.0.1; Jenkinson et al. 2012) to create heat maps in which whiter shades indicate a greater relative number of individual tractograms that contain voxels in the area; voxels with darker colors are present in relatively few individuals (Fig. S2-3).

## Supplementary Information

Below is the link to the electronic supplementary material.

Supplementary file1 (DOCX 3094 kb)

## Data Availability

Analysis code, tractography recipes, and results are available at multiple locations indicated on the lab website (www.neuroecologylab.org). Tractography recipes and results will be made available from the WIN gitlab (https://git.fmrib.ox.ac.uk/neuroecologylab/gibbon-tractography-protocols); raw data from the Digital Brain Zoo (open.win.ox.ac.uk/DigitalBrainBank/#/datasets/zoo). Analysis code is also part of the MR Comparative Anatomy Toolbox (Mr Cat; Mars et al. (2016)).
