# Supplementary material for: A map of white matter tracts in a lesser ape, the lar gibbon
Source: Brain Struct Funct. 2023 Oct 31;229(8):1839–54. doi: 10.1007/s00429-023-02709-9 (PMC11485112; doi:10.1007/s00429-023-02709-9)
Supplement: Supplementary file 1 — Supplementary file1 (DOCX 3094 kb) [file 429_2023_2709_MOESM1_ESM.docx]

**Supplementary figures**


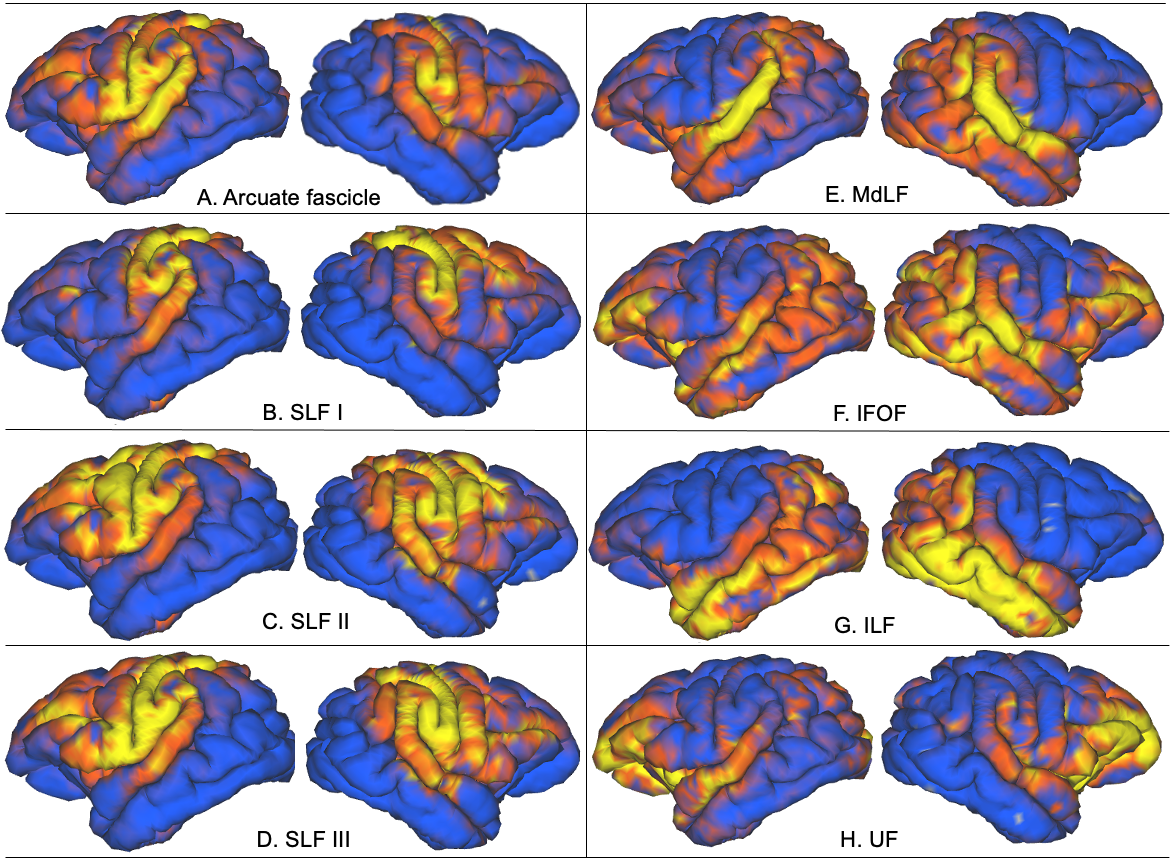


**Figure S1.** Surface projection results for major tracts: arcuate (a), SLFs I-III (b-d), MdLF (e), ILF (f), IFOF (g), and UF (h) in gibbon. Color bar indicates heat map of tractogram normalized probability values.


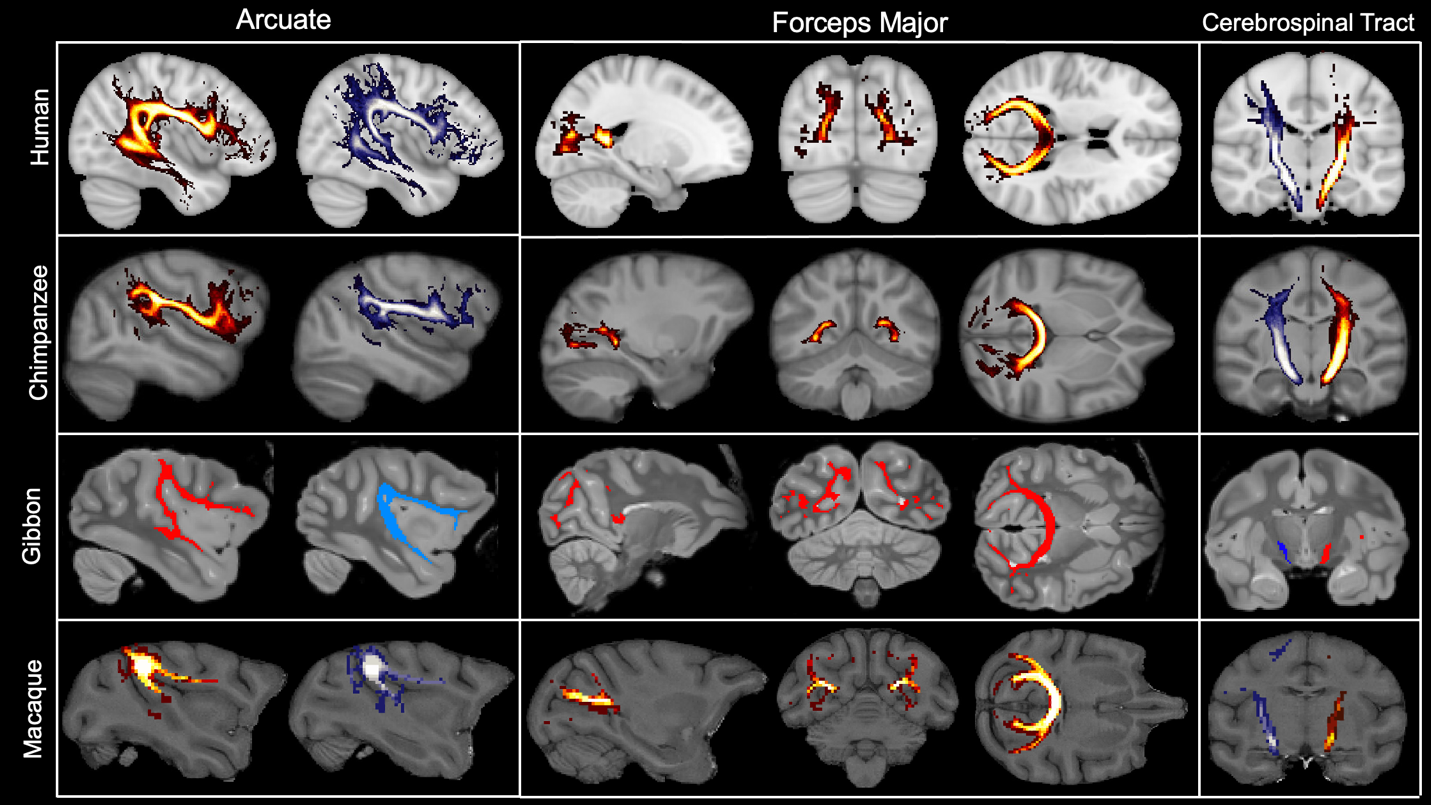


**Figure S2.** Comparative arcuate fascicle, forceps major, and corticospinal tract tractography results for human, chimpanzee, and macaque presented alongside the lar gibbon. Heat maps in human, chimpanzee, and macaque indicate the relative frequency of the presence of a particular voxel within the tractogram across each species sample. Blue-white tracts are left hemisphere, red-yellow tracts are right hemisphere.


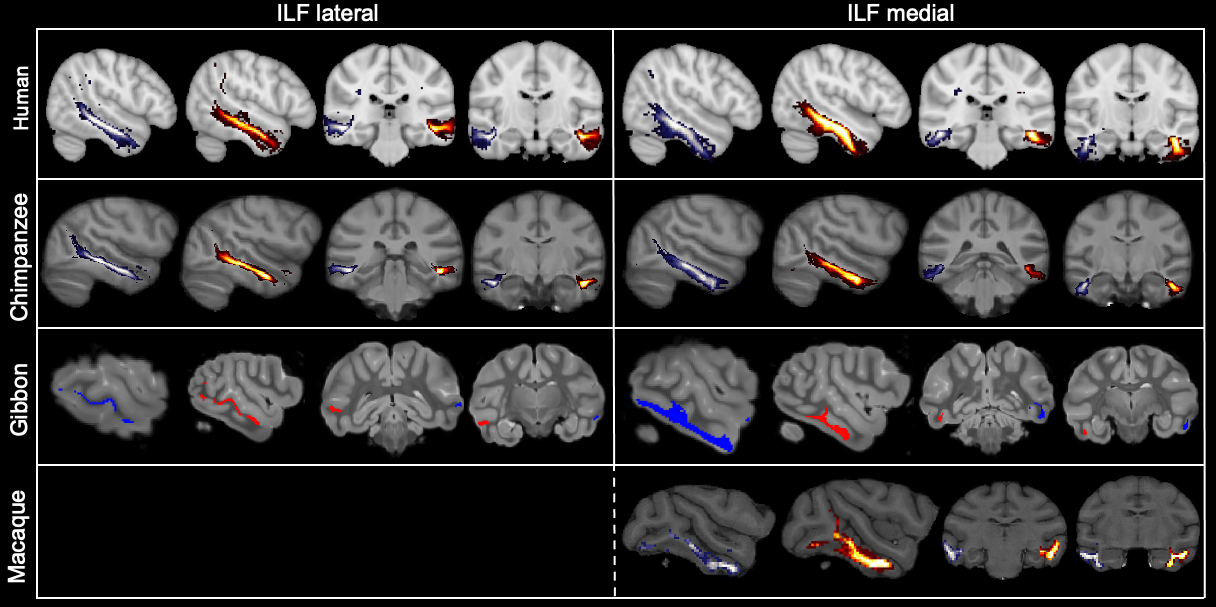


**Figure S3.** Comparative ILF tractography results for human, chimpanzee, and macaque presented alongside the lar gibbon. Heat maps in human, chimpanzee, and macaque indicate the relative frequency of the presence of a particular voxel within the tractogram across each species sample. Blue-white tracts are left hemisphere, red-yellow tracts are right hemisphere.
